# Therapeutic effects of extracorporeal shock wave therapy on patients with spastic cerebral palsy and Rett syndrome: clinical and ultrasonographic findings

**DOI:** 10.1186/s13023-023-03010-y

**Published:** 2024-01-03

**Authors:** Ting-Yu Su, Yu-chi Huang, Jih-Yang Ko, Yi-Jung Hsin, Min-Yuan Yu, Pi-Lien Hung

**Affiliations:** 1 Department of Pediatric Neurology, Kaohsiung Chang Gung Memorial Hospital and Chang Gung University College of Medicine, No. 123, Dapi Rd., Niaosong Dist., Kaohsiung City, 833 Taiwan; 2 Department of Physical Medicine and Rehabilitation, Kaohsiung Chang Gung Memorial Hospital and Chang Gung University College of Medicine, Kaohsiung City, Taiwan; 3 Department of Orthopedic Surgery, Kaohsiung Chang Gung Memorial Hospital and Chang Gung University College of Medicine, Kaohsiung City, Taiwan; 4 Department of Rehabilitation, Kaohsiung Chang Gung Memorial Hospital and Chang Gung University College of Medicine, Kaohsiung City, Taiwan; 5 Center for Shockwave Medicine and Tissue Engineering, Kaohsiung Chang Gung Memorial Hospital and Chang Gung University College of Medicine, Kaohsiung City, Taiwan

**Keywords:** Extracorporeal shockwave therapy, Cerebral palsy, Rett syndrome, Modified ashworth scale (MAS), Gross motor function measure 88 (GMFM-88), Acoustic radiation force impulse (ARFI)

## Abstract

**Background:**

Extracorporeal shock wave therapy (ESWT) is reportedly effective for improving spasticity and motor function in children with cerebral palsy (CP). Because late-stage Rett syndrome has a similar presentation, this study aimed to investigate the effects of ESWT on these two diseases.

**Material and Methods:**

Patients diagnosed with spastic CP and Rett syndrome received 1500 impulses of ESWT at 4 Hz and 0.1 mJ/mm^2^, on their spastic legsonce weekly for a total of 12 weeks. Outcomes were assessed before and 4 and 12 weeks after ESWT. Clinical assessments included the Modified Ashworth Scale (MAS), passive range of motion (PROM), and Gross Motor Function Measure 88 (GMFM-88). Ultrasonographic assessments included muscle thickness, acoustic radiation force impulse (ARFI), and strain elastography.

**Results:**

Fifteen patients with CP and six with Rett syndrome were enrolled in this study. After ESWT, patients with CP showed significant clinical improvement in the MAS (*P* = 0.011), ankle PROM (*P* = 0.002), walking/running/jumping function (*P* = 0.003), and total function (*P* < 0.001) of the GMFM-88. The patients with Rett syndrome showed improved MAS scores (*P* = 0.061) and significantly improved total gross motor function (*P* = 0.030). Under ARFI, patients with CP demonstrated decreased shear wave speed in the gastrocnemius medial head (*P* = 0.038). Conversely, patients with Rett syndrome show increased shear-wave speeds after ESWT.

**Conclusion:**

Our study provides evidence that a weekly course of low-dose ESWT for 12 weeks is beneficial for children with both CP and Rett syndrome, with the clinical effects of reducing spasticity and improving the gross motor function of the lower limbs. The ARFI sonoelastography reveals improvement of muscle stiffness in patients with CP after ESWT, but deteriorated in patients with Rett syndrome. The diverse therapeutic response to ESWT may be caused by the *MECP2* mutation in Rett syndrome, having a continuous impact and driving the pathophysiology differently as compared to CP, which is secondary to a static insult.

*Trial registration* IRB 201700462A3. Registered 22March 2017, https://cghhrpms.cgmh.org.tw/HRPMS/Default.aspx.

**Supplementary Information:**

The online version contains supplementary material available at 10.1186/s13023-023-03010-y.

## Background

Cerebral palsy (CP) is a motor impairment caused by damage to the developing brain. Preterm and very low birth weight babies have the greatest risk of developing CP. Other common causes of CP include intrauterine growth restriction and infection, and intracranial hemorrhage [1]. Spasticity is a common finding in children with upper motor neuron syndrome associated with CP. The management of children with CP requires a multidisciplinary team to meet the medical, social, psychological, educational, and therapeutic needs.

Rett syndrome is a disorder caused by mutations in X-linked methyl-CpG-binding protein 2 (*MECP2*), with its late-stage neuromotor symptoms mimicking those of CP. Most patients have a history of normal early development, followed by a period of regression and hand apraxia. Their life course can be separated into four stages: stagnation (age 6–18 months), rapid regression (age 1–4 years), pseudostationary (age 2 years–potential life), and late motor deterioration (age 10 years–life). Characteristic symptoms include a decline in motor skills, repetitive hand movements, loss of acquired speech, breathing irregularities, and seizures [2, 3].

Spasticity is a critical symptom of CP and late-stage Rett syndrome. Current therapies for spasticity include botulinum toxin injections, oral antispastic drugs, intrathecal baclofen injections, selective dorsal rhizotomies, and deep brain stimulation. Although these treatment modalities have been widely used in patients with CP, their treatment effects are usually subtle and may not be obvious. Regarding patients with Rett syndrome, few studies have emphasized the management of spasticity, with only one case reporting successful management with intrathecal baclofen [4].

Extracorporeal shock wave therapy (ESWT) was first applied to patients in 1980 for the management of nephrolithiasis and later successfully employed for many orthopedic diseases such as nonunion of long bone fracture, plantar fasciitis, calcifying tendinitis of the shoulder, several inflammatory tendon diseases, myofascial pain syndrome, and treatment of spasticity after stroke [5–8].The therapeutic effect of ESWT in patients with spastic CP has shown favorable results in literature reviews [9–12]. However, its application in treating spasticity in Rett syndrome has never been reported.

Classical spasticity is thought to increase stiffness through an overactive velocity-dependent stretch reflex. Spasticity is diagnosed using the 5-point Modified Ashworth Scale (MAS), which requires no equipment; however, it is subjective and varies widely among muscle groups [13].Various biomechanical changes within the skeletal muscle limit the validity and reliability of the MAS for evaluating spasticity associated with CP [14, 15]. For example, the main limitation of spasticity assessment using the MAS is its inability to distinguish between neural and non-neural components. Therefore, other biomechanical measures that provide reliable quantitative information are required for routine clinical use.Gross Motor Function Measure (GMFM-88) is a clinical scale that separates gross motor functions into five fields: Lying and Rolling, Sitting, Crawling and Kneeling, Standing, and Walking/Running/Jumping [16]. It is widely used to evaluate the gross motor function in patients with neuromuscular disorders and CP [17, 18].

Recently, ultrasound elastography techniques have emerged as a promising tool for the evaluation of the mechanical properties of tissues, including skeletal muscle stiffness. It involves the principle of applying stress or force toward tissue, produced by external mechanical compression, vibration, or ultrasound “push” beam, and measuring the subsequent tissue deformation. Strain elastography, acoustic radiation force impulse imaging (ARFI), and shear-wave elastography are the three main techniques used to evaluate skeletal muscles [19–22]. Few studies have investigated the utility of semi quantitative strain elastography in pediatric patients with CP as well as its feasibility for evaluating the severity of muscle stiffness and treatment effects [23–25]. In addition, ARFI, which depicts tissue displacement induced by radiation force within a small region of interest, has been used to evaluate muscle stiffness in patients with CP [26–28]. Shear-wave elastography shows good agreement in both phantoms and tissues, and is suitable for objectively quantifying muscle stiffness for individual muscles [29–31].

Based on the clinical and ultrasonographic outcomes mentioned above, this study aimed to investigate the therapeutic effects of ESWT in Taiwanese children with CP and Rett syndrome.

## Material and methods

### Patient enrollment

**Fig. 1:**
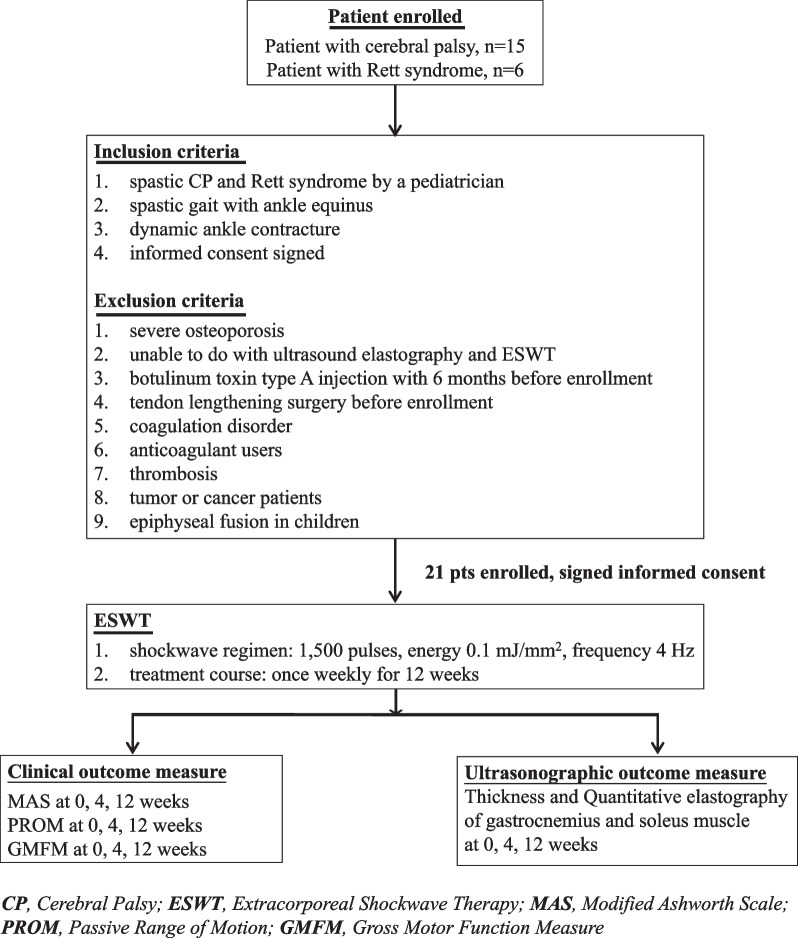
The illustrative presentation of the study design for ESWT therapy (CP: cerebral palsy, ESWT: extracorporeal shock wave therapy, pts: patients, MAS: Modified Ashworth Scale, PROM: passive range of motion, GMFM: Gross Motor Function Measure.)

This prospective study was conducted from January2017 to September 2022. Patients with spastic CP and Rett syndrome were recruited from the pediatric outpatient clinic at the Kaohsiung Chang Gung Memorial Hospital. The inclusion criteria were as follows: (1) either diagnosis of spastic CP confirmed by pediatric neurologists and physiatrists, or diagnosis of Rett syndrome made by pediatric neurologists and the pathogenic variants confirmed by a geneticist, (2) spastic gait with ankle equines, (3) dynamic ankle contracture, and (4) signed informed consent. The exclusion criteria included: (1) severe osteoporosis, (2) inability to undergo ultrasound elastography and ESWT, (3) botulinum toxin type A injection administered six months before enrollment, (4) tendon-lengthening surgery before enrollment, (5) coagulation disorder, (6) anticoagulant users, (7) thrombosis, (8) patients with tumor or cancer, and (9) epiphyseal fusion in children. The study was supervised by the Institutional Review Board (IRB no: 201700462A3). All guardians of the study participants provided informed consent. The study protocol is illustrated in Fig. 1.

### ESWT application

**Fig. 2:**
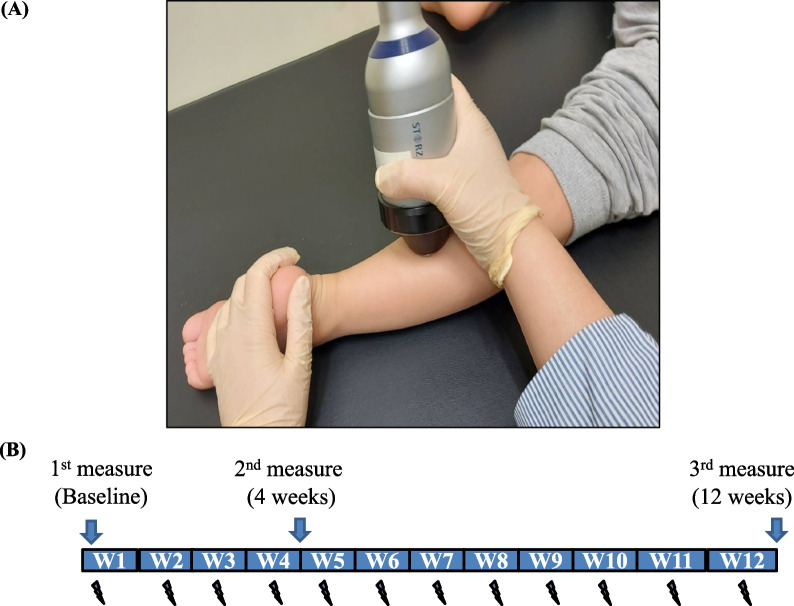
**A** The ESWT stimulating site and **B** The time course of ESWT (lightening) and outcome measurement

Patients underwent low-intensity ESWT on the spastic legs generated by an electromagnetic type machine, the Storz Medical Extracorporeal Shock Wave Therapy System^®^ (Storz Medical AG, Tägerwilen, Switzerland). The pressure pulses were focused on the midpoint between the popliteal fossa and ankle, at the conjunction point of the soleus muscle and the two heads of the gastrocnemius muscle (GCM) (Fig. 2A).The pulses were mainly applied to the middle of the muscle belly under ultrasound guidance. A total of 1,500 pulses were delivered to each leg once weekly, with the following parameters: energy flux density, 0.1 mJ/mm^2^; repetition frequency, 4 Hz; penetration depth, 15 mm; focal zone, 0–30 mm; and therapeutic effective zone, 0–90 mm. The treatment course lasted 12 weeks (Fig. 2B).

### Outcome measurement

Outcome assessments were performed before ESWT, and 4 and 12 weeks after ESWT (Fig. 2B). A licensed occupational therapist performed the clinical assessment using the Modified Ashworth Scale (MAS) [32], passive range of motion (PROM), and Gross Motor Function Measure (GMFM-88) [16]. Ultrasonography was used to measure muscle thickness, ARFI, and strain elastography of the medial head of the gastrocnemius muscle (GCM-M), lateral head of the gastrocnemius muscle (GCM-L), and soleus muscle (SOL).

### Clinical measurements

Clinical assessments were performed by an occupational therapist. The spasticity of the ankle plantar-flexormuscles was measured as the degree of resistance to passive movement using the MAS. The MAS classifies five degrees of hypertonia from 0 to 4, with higher scores representing higher muscle tone, degree 0 indicating no increase in muscle tone, and degree 4 representing affected parts rigid in flexion or extension [32]. The PROM of the ankle was assessed by measuring the angles from maximum plantar flexion to maximum dorsiflexion using a goniometer. The GMFM-88 comprises eighty-eight precise actions in the five fields, and the occupational therapist evaluated the patients’ ability to complete them by assigning a score from 0 to 3, with 0 indicating not able to perform an action and 3 indicating the ability to fully complete it [16].

### Real-time sonoelastography (RTS)

**Fig. 3:**
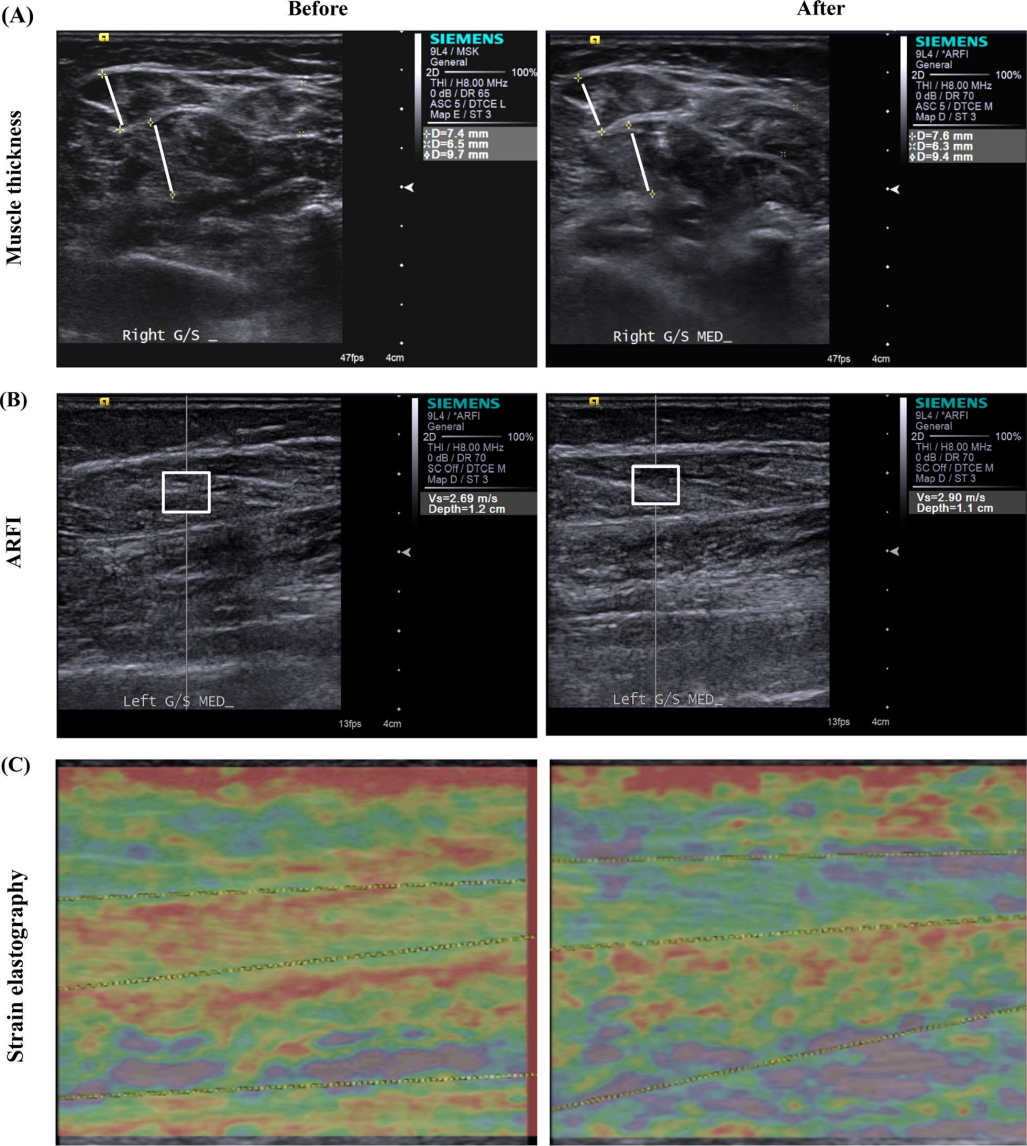
The ultrasonographic images recorded before and after ESWT. **A** muscle thickness, **B** ARFI, and **C** strain elastography (ARFI: Acoustic Radiation Force Impulse)

Ultrasonography and RTS were performed by a blinded physiatrist, who did not know the diagnosis of a subject or the results of his/her clinical measurements, using a commercially available ultrasound system (ACUSON S2000, Siemens Medical Solutions USA, Inc.) with a linear probe (9L4) before and after the 4th and the 12th week of ESWT. Ultrasonographic scans were performed repeatedly at a fixed point on the two heads of the GCM and SOL between the two reference points. One reference point was located at the proximal third of the longitudinal line from midway between the medial and lateral malleoli to midway between the medial and lateral epicondyles. The other reference point was located at the medial end of the transverse line, perpendicular to the point on the longitudinal line. Muscle thickness was defined as the distance from the superficial to the deep aponeurosis of a targeted muscle and measured under a transverse view (Fig. 3A).

ARFI was measured under a longitudinal view. A region of interest, including the muscle fascicles with clearly demarcated linear hyperechoic strands corresponding to the fibroadipose septa (i.e., perimysium) and normal surrounding tissue, was selected under ultrasound guidance. The shear wave speed within a region of interest was directly generated by the ultrasound system (Fig. 3B).

Strain elastography produced a color-coded graphic representation of muscle stiffness, with blue indicating softness, green indicating intermediate stiffness, and red indicating prominent stiffness; the results of the elastography index were generated by the ultrasound system (Fig. 3C) [19–21, 26–28].

### Statistical analysis

For the clinical outcomes of MAS and PROM and the ultrasonographic outcomes of muscle thickness, ARFI, and strain elastography, the data from the left and right legs were calculated together (N = 30 in the CP group and N = 12 in the Rett group). In the GMFM-88, each patient was considered as one subject (N = 15 in the CP group and N = 6 in the Rett group). All statistical analyses were performed using SPSS ver.22 statistical software (IBM Corp., Armonk, NY, USA). Friedman's two-way analysis of variance was used to analyze the data at the three time points of outcome measurement during the ESWT course. Comparison of the baseline scores between the CP and Rett groups was performed using the Mann–Whitney U test. The alternation of scores from baseline to 4 and 12 weeks after ESWT was compared using a generalized estimating equation (GEE). Statistical significance was set at *P* < 0.05.

## Results

### Patient profile

Fifteen children with CP and six with Rett syndrome were enrolled in this study. Among the patients with CP, six were male and nine were female (male-to-female ratio = 6:9); all patients with Rett syndrome were female. The mean age was 7.8 ± 6.0 years (ranged 3–21 years) in the CP group and 14.5 ± 2.9 years (ranged 5–22 years) in the Rett group. The median baseline GMFM-88 scores were 23.42 for the CP group and 24.85 for the Rett group. There were no significant differences in age, sex, body weight, or baseline GMFM-88 scores between the two groups (Table 1). No side effects, except a mild painful sensation, were observed by the technician or reported by the patients during or after treatment.

**Table 1:** Demographic data of children with spastic cerebral palsy and Rett syndrome

No.	Age (years) (Mean ± SEM)	*P*^*a*^	Gender (M: F)	*P*^*b*^	Body weight (kg) (Mean ± SEM)	*P*^*c*^	Baseline GMFM (Median)	*P*^*d*^
CP	7.8 ± 1.5	0.079	6: 9	0.123	21.9 ± 3.2	0.938	23.42	0.087
1	9		F		27.0		0.00	
2	5		M		16.4		23.42	
3	3		M		9.5		8.33	
4	4		F		16.6		54.33	
5	8		M		41.3		92.87	
6	5		F		17.7		18.49	
7	4		F		9.1		9.39	
8	3		F		11.2		80.94	
9	3		M		11.5		16.27	
10	6		M		15.4		70.42	
11	7		M		20.9		99.44	
12	7		F		15.3		12.80	
13	21		F		39.6		3.53	
14	20		F		48.0		36.00	
15	15		F		29.0		60.47	

Rett	14.5 ± 2.9		0: 6		29.3 ± 3.3		24.85	
1	18		F		37.0		15.11	
2	19		F		28.0		18.55	
3	17		F		41.6		70.58	
4	6		F		20.0		42.09	
5	22		F		31.0		13.63	
6	5		F		18.0		31.14	

P^a^: CP_age_ versus Rett_age_; P^b^: CP_gender_ versus Rett_gender_; P^c^: CP_body weight_ versus Rett_body weight_, P^d^: CP_GMFM_ versus Rett_GMFM_

*CP* Cerebral palsy, *RTT* Rett syndrome, *GMFM* Gross motor function measure, *SEM* Standard error of the mean

### Clinical measurement results

#### Modified ashworth scale (MAS)

The baseline MAS score of the Rett group was significantly higher than that of the CP group (*P*^*b*^ = 0.003), indicating that the baseline spasticity in the Rett group was more severe than that in the CP group. Patients with CP showed significantly decreased MAS scores after ESWT than at baseline (*P*^*a*^ = 0.011). There was also a trend toward improvement in the Rett group; however, the difference was not statistically significant (*P*^*a*^ = 0.061). There was no significant difference in the alteration of MAS score between the two groups after 12 weeks of ESWT (*P*^*c*^ = 0.930) (Table 2).

**Table 2:** Clinical measurement results at baseline and after 4 weeks and 12 weeks of ESWT

	RETT measurements				CP measurements				Comparison of two groups	
Variables (Mean ± SEM)	1 (Baseline)	2 (4 weeks)	3 (12 weeks)	*p*^a^	1 (Baseline)	2 (4 weeks)	3 (12 weeks)	*p*^a^	*p*^b^	*p*^c^
*MAS of lower extremity flexor*										
	4.00 ± 0.26	3.50 ± 0.43	3.33 ± 0.49	0.061	2.20 ± 0.30^**§**^	1.63 ± 0.31	1.50 ± 0.28^**§**^	0.011*	0.003**	0.930
*Ankle PROM (º)*										
	57.08 ± 3.68	58.33 ± 3.07	58.33 ± 3.07	0.135	47.50 ± 3.88	55.00 ± 2.20	55.50 ± 2.07	0.002**	0.177	0.020*
*GMFM-88 Variables Score (%)*										
Lying and Rolling	68.30 ± 11.63	68.63 ± 11.49	68.95 ± 11.36	0.368	66.93 ± 8.94	67.45 ± 8.83	68.24 ± 8.70	0.305	0.936	0.586
Sitting	53.33 ± 11.46	53.33 ± 11.46	53.33 ± 11.46	1.000	46.33 ± 10.81	49.44 ± 10.76	49.00 ± 10.71	0.079	0.410	0.091
Crawling and Kneeling	0.00 ± 0.00	0.00 ± 0.00	0.00 ± 0.00	1.000	29.21 ± 11.08	30.16 ± 11.11	30.16 ± 11.11	0.135	0.079	0.153
Standing	20.94 ± 13.08	21.79 ± 13.53	22.22 ± 13.93	0.156	29.40 ± 9.70	31.11 ± 9.90	31.79 ± 10.04	0.076	0.866	0.440
Walking/Running/Jumping	16.67 ± 11.08	16.90 ± 11.31	17.36 ± 11.51	0.156	23.70 ± 8.69	25.46 ± 9.05	27.69 ± 9.44	0.003**	0.968	0.083
Total	31.85 ± 8.93	32.13 ± 9.04	32.37 ± 9.13	0.030*	39.11 ± 8.86^**§**^	40.73 ± 8.93	41.37 ± 9.06^**§**^	0.000***	0.938	0.030*

*p* value: *< 0.05, **< 0.01, ***< 0.001

*p*^a^: Comparison between three assessing time points, Friedman's two-way analysis of variance

*p*^b^: Comparison between the group of Rett syndrome and CP at baseline, Mann–Whitney Test

*p*^c^: Comparison of the alteration from baseline to 12 weeks between the group of Rett syndrome and CP, GEE

^§^: Comparison between baseline and 12th week, *p* value: < 0.05, multiple comparison of Friedman's two-way analysis of variance

*CP* Cerebral palsy, *RETT* Rett syndrome, *SEM* standard error of the mean, *MAS* Modified Ashworth Scale, *PROM* Passive range of motion, *GMFM* Gross motor function measure

#### Passive range of motion (PROM)

There was no significant difference in the baseline PROM assessment between the two groups (*P*^*b*^ = 0.177). The PROM angle increased significantly after 12 weeks of ESWT compared to the baseline assessment in the CP group (*P*^*a*^ = 0.002);however, there was no significant change in the PROM angle for the Rett group after ESWT (*P*^*a*^ = 0.135). The CP group showed a significantly greater improvement in PROM after 12 weeks of ESWT than the Rett group (*P*^*c*^ = 0.020) (Table 2).

#### Gross motor function measure-88 (GMFM-88)

**Fig. 4:**
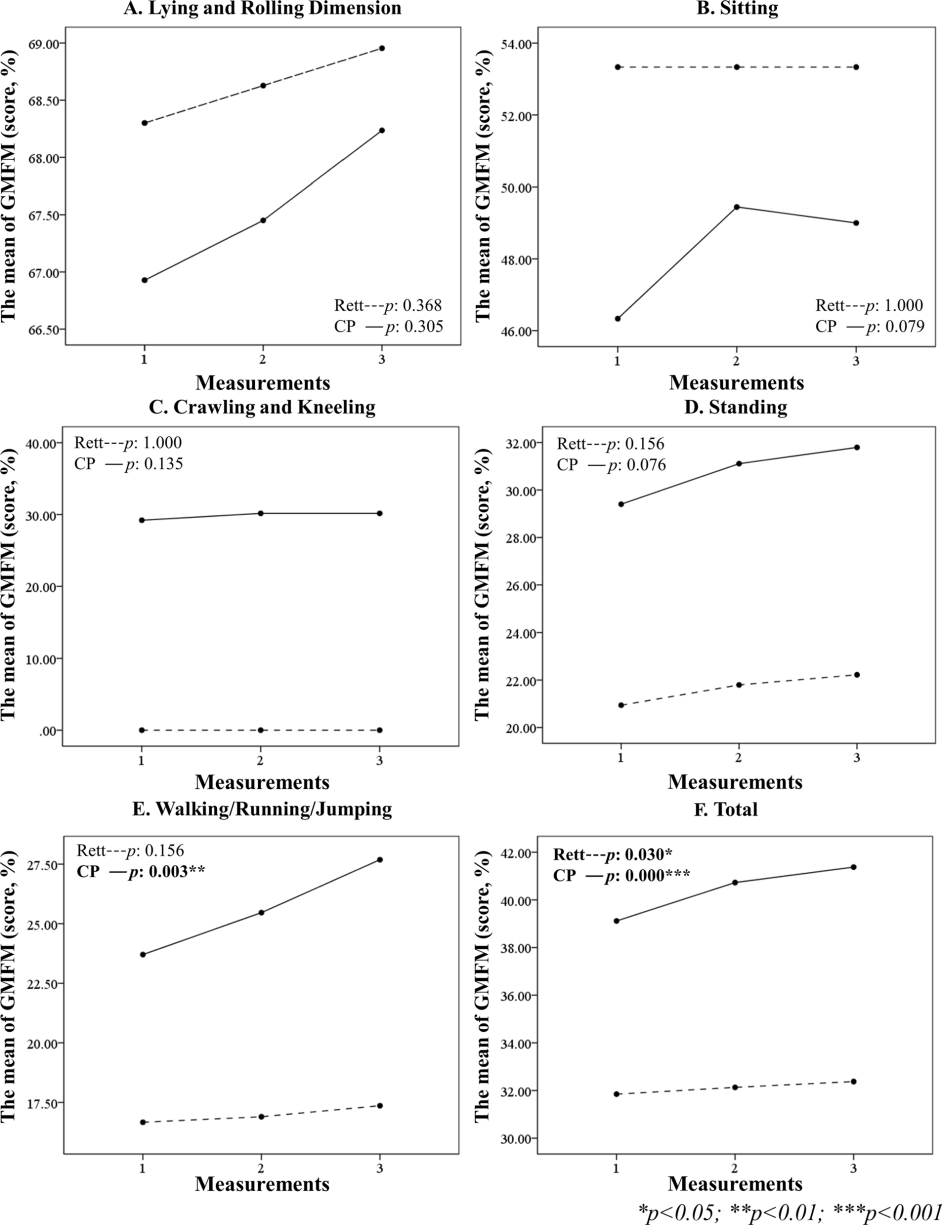
The GMFM-88 of lower extremities before and after ESWT in patients with CP and Rett syndrome. The walking/running/jumping function score increased significantly in the CP group (**E**), and the total function score increased significantly in both groups (**F**). (CP: cerebral palsy, GMFM: Gross Motor Function Measure)

In the GMFM-88 assessment of the lower limbs, no significant difference in the baseline motor function was found between the two groups. After 12 weeks of ESWT, there was a significant improvement in the total GMFM-88 score in both the CP (*P*^*a*^ < 0.001) and Rett groups (*P*^*a*^ = 0.030). In addition, there was a significant improvement in walking/running/jumping function in the CP group (*P*^*a*^ = 0.003), but not in the Rett group (*P*^*a*^ = 0.156). Comparing the alterations after 12 weeks of ESWT, the CP group showed significantly greater improvement than the Rett group in the total GMFM-88 score (*P*^*c*^ = 0.030) (Table 2, Fig. 4).

### Ultrasonographic measurement results

#### Muscles thickness

There was no significant difference in the baseline muscle thickness between the two groups. After ESWT, the CP and Rett groups showed no significant alterations in muscle thickness in the GCM-M, SOL, or GCM-L (Table 3).

**Table 3:** Ultrasonographic measurement results at baseline and after 4 weeks and 12 weeks of ESWT

	RETT measurement				CP measurement					Comparison of two groups	
Variables (Mean ± SEM)	1 (Baseline)	2 (4 weeks)	3 (12 weeks)	*p*^a^		1 (Baseline)	2 (4 weeks)	3 (12 weeks)	*p*^a^	*p*^b^	*p*^c^
*Muscle thickness (mm)*											
GCM-M	10.12 ± 0.41	11.8 ± 0.84	11.28 ± 0.77	0.260		10.24 ± 1.14	11.29 ± 0.95	11.52 ± 0.90	0.145	0.726	0.886
SOL	11.31 ± 1.17	10.78 ± 0.81	9.77 ± 0.88	0.154		11.17 ± 1.19	10.60 ± 0.85	10.58 ± 0.78	0.158	0.938	0.426
GCM-L	8.41 ± 0.87	7.26 ± 0.71	8.06 ± 0.55	0.223		7.64 ± 0.69	8.13 ± 0.68	7.93 ± 0.60	0.617	0.586	0.361
*ARFI shear wave speed (m/s)*											
GCM-M	1.46 ± 0.35^**§**^	1.96 ± 0.23	2.42 ± 0.14^**§**^	0.030*		2.59 ± 0.13	2.40 ± 0.14	2.35 ± 0.11	0.038*	0.010*	0.000***
SOL	1.24 ± 0.18^**§**^	1.71 ± 0.19	1.97 ± 0.13^**§**^	0.030*		2.00 ± 0.12	1.87 ± 0.10	1.87 ± 0.09	0.189	0.008**	0.000***
GCM-L	1.50 ± 0.24	1.93 ± 0.21	2.11 ± 0.11	0.115		2.29 ± 0.10	2.21 ± 0.14	2.20 ± 0.11	0.766	0.009**	0.000***

*p* value: *< 0.05, **< 0.01, ***< 0.001

*p*^a^: Comparison between three assessing time points, Friedman's two-way analysis of variance

*p*^b^: Comparison between the group of Rett syndrome and CP at baseline, Mann–Whitney Test

*p*^c^: Comparison of the alteration from baseline to 12 weeks between the group of Rett syndrome and CP, GEE

^§^: Comparison between baseline and 12th week, *p* value: < 0.05, multiple comparison of Friedman's two-way analysis of variance

*CP* Cerebral palsy, *RETT* Rett syndrome, *SEM* Standard error of the mean, *GCM-M* Gastrocnemius medial head, *SOL* Soleus muscle, *GCM-L* Gastrocnemius lateral head

#### Acoustic radiation force impulse (ARFI)

Under ARFI elastography, the baseline shear wave speed was significantly higher in the CP group than in the Rett group in all three muscles (*P*^*b*^ = 0.010 in the GCM-M, 0.008 in the SOL, and 0.009 in GCM-L), indicating a more prominent baseline muscle stiffness in patients with CP. After 12 weeks of ESWT, a significant decrease in the shear wave speed was observed in the GCM-M of the CP group (*P*^*a*^ = 0.038), indicating an improvement in muscle stiffness. Conversely, the shear wave speed of the Rett group increased significantly in the GCM-M and SOL groups (*P*^*a*^ = 0.030 and 0.030, respectively), indicating progressive muscle stiffness despite ESWT. There was a significantly greater decrease in shear wave speed in all three muscles in the CP group than in the Rett group after 12 weeks of ESWT (*P*^*d*^ < 0.001 in GCM-M, < 0.001 in SOL, and < 0.001 in GCM-L) (Table 3).

#### Strain elastography

The baseline elastography index of green and blue in the GCM-M was significantly higher in the Rett group than in the CP group, indicating less severe baseline muscle stiffness in patients with Rett syndrome, which was compatible with the ARFI results. After ESWT, the alteration of the elastography index in both the CP and Rett groups was unremarkable, with random changes in the red, green, and blue colors (Additional file 1: Table S1).

## Discussion

To the best of our knowledge, this is one of the first trials to evaluate the application of ESWT on patients with Rett syndrome. Moreover, previous studies on ESWT in patients with CP focused on clinical outcomes but seldom discussed ultrasonographic assessments [33]. In this study, we used both clinical scores and ultrasound to evaluate the therapeutic effects. Our results showed that after 12 weeks of low-intensity ESWT, both patients with CP and those with Rett syndrome showed clinical improvement in total gross motor function (evaluated using the GMFM-88). In addition, patients with CP had improved spasticity (evaluated using MAS) and range of motion in ankles. On ultrasonographic assessment, patients with CP showed improved muscle stiffness after ESWT. Conversely, patients with Rett syndrome showed signs of increased muscle stiffness (illustrated by ARFI).

Our study demonstrated a significant decrease in the MAS score of the lower limb flexors after ESWT in the CP group and a trend of decrement in the Rett group. In addition, the CP group showed significantly increased PROM of the ankles after ESWT. Randomized controlled trials in adults with stroke have shown similar effects of ESWT with significantly decreased MAS; however, the results differ regarding the alteration of PROM [34, 35]. Previous studies on patients with CP have revealed compatible results of significant improvements in both the MAS and PROM of the ankle [10, 12, 17, 18, 36, 37]. In addition, ESWT has been shown to be more effective than conventional physical therapies [38], and the combination of ESWT with botulism toxin A injection provided better improvement in MAS and PROM than botulism injection alone [6, 39].

After ESWT, our study showed a significant improvement in walking/running/jumping function in the CP group and in total function in both groups. The gross motor function of the lower extremities in adults with stroke have shown significant improvement after ESWT [34, 35].In addition, patients with CP have shown significantly increased GMFM-88 scores after ESWT, especially in the Standing and Walking/Running/Jumping functions [17, 18]. To summarize, most trials have concluded that ESWT has promising clinical effects in patients with CP and stroke. Moreover, our study revealed its benefits in improving gross motor function and spasticity in patients with Rett syndrome, although it was more effective in patients with CP (Additional file 2).

However, the changes in muscle thickness were not consistent with those of previous studies. Although muscle thickness and spasticity decreased after ESWT in adults with stroke [34], there were no significant changes in post-therapeutic muscle thickness in our study. In our hypothesis, this may have been caused by the rapid muscle growth in our pediatric patients. Previous studies on ESWT in patients with CP did not discuss the alterations in muscle thickness.

ARFI has been used as a non-invasive and feasible method to evaluate the muscle stiffness of patients with CP [23, 26] and the effects of botulinum toxin injection [27, 40]. In our study, the CP group showed a significantly decreased shear wave speed after ESWT in the GCM-M but not in the SOL or GCM-L. We supposed that this was caused by the penetration of the shock wave and depth of the muscles. In this study, we used a hand piece with a short penetration depth of 15 mm and a focal zone of 30 mm; thus, the superficial muscle was more vulnerable to shockwave therapy. Picelli et al. used sonography to measure muscle stiffness and revealed a significantly greater reduction in muscle hardness percentage, which indicated a greater reduction in muscle stiffness when applying additional ESWT to conventional botulinum injection therapy for patients with CP [33]. Their conclusion corroborated our finding of decreased shear wave velocity on ARFI elastography after ESWT in patients with CP. The strain elastography data were highly related to the pressure applied by the manipulator to the targeted tissue. Therefore, the unremarkable elastography index results were likely caused by the lack of cooperation and voluntary movement of our pediatric patients.

In our result, the baseline MAS is significantly higher in Rett group than CP group, however, the baseline shear wave velocity in ARFI imaging is significanly higher in CP group than Rett group. We provided the possible explanation for the discrepancy results herein. In ARFI, an ultrasound transducer generates a push beam to apply stress, after which the same transducer measures the tissue displacement along the push beam. ARFI measurements are more reliable than traditional strain elastography measurements because tissue displacement with ARFI is caused by fixed ultrasound waves, rather than by tissue compression by the sonographer and thus was less likely interfered by manipulative error. MAS is a clinical measurement performed by an occupational therapist, who measures the degree of resistance to passive movement of the ankle plantar-flexor muscles and classifies the spasticity into five scores. In addition, the ARFI indicated microscopic muscle stiffness in one single muscle while the MAS referred to gross muscle tone of ankle plantar-flexors. From this point of view, ARFI elastography and MAS are quite different tool for assessing muscular spasticity. We also demonstrated the similar result as previous research [41], which showed a weak correlation between the clinical MAS and the shear wave velocity of biceps brachii muscle in ARFI imaging. Thus, these two parametersdo not necessarily have positive correlation in clinical application.

In the Rett group, despite clinical improvement of muscle spasticity and gross motor function in MAS and GMFM-88 assessment, the muscle stiffness seemed to get worse after ESWT in the ARFI elastography. We can provide the possible explanation for the contradictory results. First, being a neurodevelopmental disorder, patients with Rett syndrome have regressive course in motor functions, spasticity, and muscle stiffness time-by-time, which may be the reason why the shear wave velocity in ARFI imaging was progressively higher after ESWT. Second, the MAS was not positively correlated with ARFI just as we stated. Thus, patients with Rett syndrome showed decrement in MAS score after ESWT may not lead to decrease shear wave velocity in ARFI imaging. We gave a conclusion that ESWT ameliorated the clinical spasticity, but not muscle stiffness in patients with Rett syndrome. However, the disappointed result may contribute to the low shock wave energy used in our study. Beside our study, sonoelastography has also been utilized in a few studies to monitor muscle stiffness in patients with genetic entity such as Duchenne muscular dystrophy [42]. In the future, it might be a useful tool for monitoring the precise microstructural alterations in patients with a genetic etiology.

In the late stages, the motor function and spasticity of Rett syndrome mimic those of CP. However, whether the mechanisms underlying spasticity in CP and Rett syndrome are similar remain unknown. In this study, we attempted to manage muscular spasticity using ESWT in the same setting and investigated the therapeutic response in these two diseases. Our data revealed that the therapeutic responses to ESWT between CP and Rett was quite different. After ESWT, there was a significant improvement in spasticity, ankle joint range of motion, and gross motor function in the CP group compared with the Rett group. These results indicated that patients with CP were more responsive to ESWT than those with Rett syndrome. A possible explanation could be that the baseline spasticity was more severe in the Rett group, which needed a higher shock wave energy than the CP group. In addition, we supposed that patients with CP and Rett syndrome share different mechanisms of muscular spasticity; therefore, they show different responses to ESWT.

Previous research revealed several possible mechanisms of ESWT reducing muscle spasticity, such as (1) by acting directly on fibrous tissue to alter the rheological properties, e.g. muscle elasticity and extensibility [43, 44], (2) by inducing nitric oxide production to reduce intramuscular connective tissue stiffness [45], (3) by inhibiting transmission at neuromuscular junctions and inducing degeneration of acetylcholine receptors [46], and (4) by enhancing growth of axonal regeneration followed by partial destruction [47]. The pathophysiology of spasticity and muscle tissue in Rett syndrome were rarely discussed in previous study, but generally, the brain circuitry related to hypertonia included cholinergic, dopaminergic, GABAergic, and glutaminergic pathways [48]. Among these mechanisms, anticholinergics such as Trihexylphenidyl had shown effectiveness in management of hypertonia/dystonia on patients with Rett syndrome [49] but not on those with CP [50]. This finding provided a clue that the third mechanism of ESWT mentioned above, inducing degeneration of acetylcholine receptors, may act a more important role in Rett syndrome. Animal studies on rats found a minimal requirement of energy flux densities (EFD) at 0.09 mJ/mm^2^ with total exposure of 360 mJ to inhibit the transmission in neuromuscular junction [51]; they also found the time to recovery from inhibition to be 8 weeks after ESWT [46]. Based on these findings, our current protocol of 1500 pulses with EFD at 0.1 mJ/mm^2^ might be insufficient for patients with Rett syndrome. For further quantitative study, a protocol of 3600 pulses with EFD at 0.1 mJ/mm^2^ or 4000 pulses with EFD at 0.09 mJ/mm^2^ and a post-therapeutic follow-up of at least 8 weeks should be considered.

Our study has some limitations. First, as Rett syndrome being a rare disease, the small sample size provided a limited quality of evidence; thus, a systematic review, meta-analysis, or a multicenter study with large-scale, well-designed trials is required to provide convincing conclusions. Second, our study lacked a comparison with conventional therapeutic interventions, such as physical therapy or botulinum injections. Third, the observation period provided only intra-therapeutic outcomes, but not long-term effects, after the completion of ESWT. Previous meta-analyses revealed that MAS was significantly reduced for only one month after ESWT, but spasticity-associated factors (e.g., ankle ROM) could persist for over three months and the outcomes were not dependent on shock wave doses [52, 53]. Despite the above limitations, this is a pivotal study that evaluated the application of ESWT in patients with Rett syndrome. Furthermore, the measurement of both clinical and ultrasonographic outcomes after ESWT in children could be a good reference for associated research. Being a less invasive, less painful, and more cost-effective therapeutic option than botulinum injection, the utility of ESWT on pediatric population is highly valuable.

## Conclusion

Our study provides evidence that a weekly course of low-dose ESWT for 12 weeks is beneficial for children with both CP and Rett syndrome, with the clinical effects of reducing spasticity and improving the gross motor function of the lower limbs. The ARFI sonoelastography reveals improvement of muscle stiffness in patients with CPafter ESWT, but not in those with Rett syndrome. The diverse therapeutic response to ESWT may be caused by the *MECP2* mutation in Rett syndrome, having a continuous impact and driving the pathophysiology differently as compared to CP, which is secondary to a static insult.

### Supplementary Information

**Additional file 1: Table S1.** Comparison of elastography indices before and after ESWT in patients with CP and Rett syndrome.

**Additional file 2. Table S2.** The raw data of AFRI and MAS score of all study subjects

## Data Availability

The datasets generated and/or analysed during the current study are not publicly available because most of them are unorganized scales or but are available from the corresponding author on reasonable request.

## Abbreviations

ARFI: Acoustic radiation force impulse imaging
CP: Cerebral palsy
ESWT: Extracorporeal shock wave therapy
GCM: Gastrocnemius muscle
GMFM-88: Gross motor function measure
GCM-L: Lateral head of the gastrocnemius muscle
GCM-M: Medial head of the gastrocnemius muscle
MAS: Modified Ashworth scale
PROM: Passive range of motion
SOL: Soleus muscles
