# Supplementary material for: Therapeutic effects of extracorporeal shock wave therapy on patients with spastic cerebral palsy and Rett syndrome: clinical and ultrasonographic findings
Source: Orphanet J Rare Dis. 2024 Jan 3;19:6. doi: 10.1186/s13023-023-03010-y (PMC10763338; doi:10.1186/s13023-023-03010-y)
Supplement: Supplementary file 1 — Additional file 1: Table S1. Comparison of elastography indices before and after ESWT in patients with CP and Rett syndrome. [file 13023_2023_3010_MOESM1_ESM.docx]

**Supplementary Table 1. The outcome of elastography index in strain elastography**

|  | **RETT** | | | | **CP** | | | | | Comparison | |
| --- | --- | --- | --- | --- | --- | --- | --- | --- | --- | --- | --- |
|  | Measurements | | | | Measurements | | | | | of two groups | |
| Variables  (Mean±SEM) | 1  (Baseline) | 2  (4 weeks) | 3  (12 weeks) | *p*^a^ | | 1  (Baseline) | 2  (4 weeks) | 3  (12 weeks) | *p*^a^ | *p*^b^ | *p*^c^ |
| **GCM-M (%)** | | | | | | | | | | | |
| Red | 117.40±0.96 | 124.55±2.82**^‡^** | 118.02±1.93**^‡^** | **0.042*** | | 120.47±1.35 | 119.15±0.92 | 119.44±0.96 | 0.395 | 0.119 | 0.477 |
| Green | 137.83±0.82 | 139.86±3.12 | 137.11±1.71 | 0.154 | | 131.76±1.27 | 131.53±1.25 | 132.32±1.25 | 0.751 | **0.005**** | 0.717 |
| Blue | 112.56±0.81 | 113.36±2.44 | 117.96±2.34 | **0.019*** | | 106.54±1.42 | 108.90±1.88 | 108.09±2.11 | 0.223 | **0.005**** | 0.193 |
| **SOL (%)** | | | | | | | | | | | |
| Red | 115.46±1.35 | 123.73±2.92 | 120.23±2.14 | 0.070 | | 117.66±1.70 | 117.90±1.08 | 118.57±1.18 | 0.526 | 0.533 | 0.153 |
| Green | 134.41±0.87**^§^** | 137.88±2.08 | 138.75±1.99**^§^** | **0.042*** | | 132.62±0.87**^†^** | 134.05±0.85**^†^** | 133.61±1.10 | **0.046*** | 0.243 | **0.041*** |
| Blue | 110.22±3.05 | 113.98±3.03 | 114.48±2.71 | 0.676 | | 108.18±2.04 | 107.39±1.64 | 108.21±1.75 | 0.931 | 0.640 | 0.227 |
| **GCM-L (%)** | | | | | | | | | | | |
| Red | 121.40±2.08 | 124.14±2.83 | 119.63±1.43 | 0.115 | | 121.09±1.22 | 120.94±1.28 | 120.12±0.83 | 0.232 | 0.869 | 0.753 |
| Green | 134.90±1.52**^†^** | 141.18±1.59**^†^** | 138.56±2.40 | **0.030*** | | 132.10±1.34 | 132.62±1.57 | 132.66±1.69 | 0.794 | 0.248 | 0.083 |
| Blue | 112.35±3.89 | 116.32±1.80 | 119.55±2.27 | 0.223 | | 106.32±1.53 | 108.91±2.26 | 109.27±1.97 | 0.368 | 0.216 | 0.103 |
| *p*-value: *<0.05, **<0.01, ***<0.001  *p*^a^: Comparison between three assessing time points, Friedman's two-way analysis of variance  *p*^b^: Comparison between the group of Rett syndrome and CP at baseline, Mann-Whitney Test  *p*^c^: Comparison of the alteration from baseline to 12 weeks between the group of Rett syndrome and CP, GEE  †: Comparison between the measurement of baseline and 4 weeks, *p*-value: <0.05, multiple comparison of Friedman's two-way analysis of variance  §: Comparison between the measurement of baseline and 12 weeks, *p*-value: <0.05, multiple comparison of Friedman's two-way analysis of variance  ‡: Comparison between the measurement of 4 weeks and 12 weeks, *p*-value: <0.05, multiple comparison of Friedman's two-way analysis of variance  **CP**: Cerebral palsy; **RETT**: Rett syndrome; **SEM**: standard error of the mean**; GCM-M**: gastrocnemius medial head; **SOL**: soleus muscle; **GCM-L**: gastrocnemius long head | | | | | | | | | | | |
